# Patient safety culture in dental clinics: A cross-sectional study in Afghanistan

**DOI:** 10.1371/journal.pone.0359289

**Published:** 2026-10-28

**Authors:** Safiyeh Osmani, Zahra Emrani, Saeed Akhlaghi, Amin Shahraki, Javad Moghri, Fatemeh Kokabisaghi

**Affiliations:** 1 Hariva institute of Higher Education, Herat, Afghanistan; 2 Student Research Committee, School of Health, Mashhad University of Medical Sciences, Mashhad, Iran; 3 Social Determinants of Health Research Center, Basic Sciences Research Institute, Mashhad University of Medical Sciences, Mashhad, Iran; 4 Department of Health Economics and Management Sciences, School of Health, Mashhad University of Medical Sciences, Mashhad, Iran; 5 Department of Biostatistics, School of Health, Mashhad University of Medical Sciences, Mashhad, Iran; University of the Witwatersrand Johannesburg Faculty of Health Sciences, SOUTH AFRICA

## Abstract

Healthcare safety is a major global concern since unsafe and low-quality services can lead to poor health outcomes and even harm to patients. Promoting a strong patient safety culture is a prerequisite for improving patient safety. The present study aimed to assess patient safety culture in dental clinics in Herat, Afghanistan in 2024. This cross-sectional study included 210 dental team members selected by random cluster sampling from 7 regions. The assessment tool was the Medical Office Survey on Patient Safety Culture (MOSOPS) modified for use in dentistry. A pilot study confirmed content validity and reliability (Cronbach’s alpha > 0.70). Data were analyzed using descriptive statistics, bivariate tests (t-tests and ANOVA), and multiple linear regression to examine associations with patient safety culture. The total patient safety culture score was (0.70 ± 0.10) (on a 0–1 scale), indicating a moderate level. The strongest mean scores were for organizational learning (0.78 ± 0.14) and teamwork (0.77 ± 0.17). The lowest score was observed for office processes and standardization (0.62 ± 0.13). Multiple linear regression revealed that being female (B = 0.031, p = 0.049) and holding a managerial position (B = 0.073, p < 0.001) were significantly associated with higher patient safety culture scores. Overall, patient safety culture was generally favorable, although important organizational gaps remain. Future improvement efforts should focus on standardizing office processes and strengthening information exchange with external healthcare providers. In resource-limited settings such as Afghanistan, feasible interventions including locally adapted protocols, and ongoing managerial support and supervision may further strengthen patient safety culture.

## Introduction

Medical errors represent a significant public health concern. A culture focused on system improvement and actionable solutions—rather than blame and punishment—enables healthcare organizations to learn from adverse events and improve patient outcomes [1]. Patient safety is defined as “the reduction of risk of unnecessary harm associated with health care to an acceptable minimum” [2]. Approximately two-thirds of adverse events, and the disabilities resulting from medical errors occur in low- and middle-income countries [3]. In these countries, a combination of multiple factors, such as inadequate staffing, organizational structure, overcrowding, inadequate healthcare supplies, equipment, and unsafe patient care, contribute to harm [4]. International efforts to reduce patient harm have recently made significant progress. Despite pioneering work in some healthcare settings, there has been limited systematic and consistent progress in healthcare safety globally [5]. A review of the literature by Carpenter et al. in developing and emerging countries showed that patient safety has been underdeveloped and understudied [6]. There is now increasing concern for the safety of healthcare systems. Assessing and promoting patient safety culture is recognized as a necessary step toward improving patient safety [7].

Safety is about reducing adverse health care-related events in order to reduce the risk of harm to patients. Patient safety culture is a set of values and policies shared by all professionals in an organization [8]. Various studies showed that safety culture leads to higher clinical performance and a reduction in adverse events, incidents, and errors.

Although patient safety has traditionally been a priority in hospitals, dental care also poses preventable risks to patients—including errors in diagnosis, treatment, communication, medication management, and follow-up. Unlike hospital care, dentistry is often delivered in private or ambulatory settings, where adverse events may go unidentified or unreported [9,10]. In this context, patient safety culture—encompassing communication, teamwork, and standardized processes—plays a critical role in preventing and managing safety risks [9–11].

In recent years, dental care providers have come to understand the critical importance of prioritizing patient safety, given that safety issues commonly cause patient harm and undermine the quality of care [12]. Furthermore, enhanced safety culture has been shown to correlate with more favorable patient safety outcomes [13].

Global neglect of oral healthcare services, especially in low- and middle-income countries, drives health system decline and fuels rising global health inequality [14]. Compared with other types of medical care, dentistry has been slow in addressing patient safety because 1) in dental care, severe harms are less likely; 2) it is difficult to identify and follow up on care-related incidents in the outpatient setting; 3) deficits in patient data records make it difficult to collect data; 4) most dental care occurs in private clinics, and because of commercial impact, adverse events might not be reported and 5) the lack of a widespread patient safety culture [9]. A study in Indonesia found some issues related to patient safety including limited or lack of informed consent, non-compliance with the laws, administrative negligence, poor communication, medical malpractice negligence and misdiagnosis [15]. Improving communication, encouraging reporting, and better management of care can help decrease harm to the patient [16].

Errors in diagnosis, examination, treatment, communication, and care processes, as well as accidental ingestion or inhalation of objects, have been identified as important patient safety issues in dentistry [10].

However, organizational issues such as organizational learning and management support for patient safety have received less attention [10]. A systematic review revealed that poor understanding of how patient safety in dentistry differs from other healthcare fields remains a barrier to improving patient safety in dentistry [9]. A study on patient safety culture in dental practice in the United States showed that patient safety culture needed to be improved in various aspects [11].

Patient safety culture in an organization ensures reduced adverse events. Measuring patient safety culture is a prerequisite to planning for improving safety and quality of care [8]. Few studies focused on patient safety culture in dental practice. In particular, evidence on safety culture in Afghanistan’s dental sector is lacking.

Although previous research has assessed patient safety culture in Afghan hospitals [3], evidence specific to dental settings is lacking [9–11]. This gap is important because the organizational and care-delivery characteristics of dental practice differ from those of hospital-based healthcare and may involve distinct patient safety challenges.

In Afghanistan, available evidence indicates that patient safety culture is weak. Resource shortages, high workload, and the lack of standard infrastructure are the main contributing factors to this situation [3]. This study aimed to examine patient safety culture in dental clinics located in Herat city, Afghanistan in 2024. The results of the study can help healthcare providers, medical universities, policy makers, and future researchers in promoting patient safety culture.

## Methodology

This study has a cross-sectional design. The study population included all dental practitioners and dental staff in Herat city, Afghanistan. Given the lack of a similar study in this country, the sample size was determined based on the proportion of dentists having high perceptions of patient safety culture in the study by Yansaneh et al. [11]; this proportion was chosen to determine the maximum sample size. Considering P = 50, margin of error of 0.85 and a 95% confidence, the sample size was determined at 133. Since the sampling method was cluster sampling, a correction factor of 1.5 was considered that increased the sample size to 210. To achieve this sample size, according to the study of Yansaneh et al. [11], seven regions were randomly selected from 15 regions of the city, and from each region, three dental units and five dental practitioners and qualified dental staff from each unit were chosen by simple random sampling.

Inclusion criteria included working as a dentist, dental school clinical faculty, dental student, dental hygienist, and dental clinic staff (including dental assistants, nurses, receptionists, business office staff, clinic managers, dispensary staff and patient care coordinators). Exclusion criteria were first- and second-year dental students and temporary/volunteer staff.

The research instrument was the Medical Office Survey on Patient Safety Culture (MOSOPS) questionnaire modified for dentistry, which is an extension of the Agency for Healthcare Research and Quality (AHRQ) hospital survey on patient safety culture and has been adapted to the dental office environment [11]. In addition to demographic characteristics such as age, sex, education, working hours per week, and work and management experience, the questionnaire comprised 10 safety culture dimensions; 6 overall quality items and 9 patient safety and quality issues. The dimensions included overall perceptions of patient safety and quality, organizational learning, teamwork, staff training, work pressure and pace, management support for patient safety, office processes and standardization, communication about error, communication openness, and patient care tracking/follow-up, in addition to overall quality items measuring patient-centeredness, effectiveness, timeliness, efficiency, equity, and overall rating; patient safety and quality issues; and exchanging information with other health care settings. All questions were scored on a Likert scale (strongly disagree to strongly agree) except for the communication and details, which were answered with daily, weekly, several times a year, once or twice a year, never happened in the past 12 months, don’t know, and not applicable [11].

The questionnaire went through forward and backward translation to the common languages of Afghanistan, followed by expert review and pilot testing involving 30 participants from the target population to assess the clarity and appropriateness of the translated questionnaire. Internal consistency was assessed using Cronbach’s alpha was 0.89, which is at an acceptable level, and reliability calculated using the test-retest method and ICC was 0.81.

Researchers visited the participating dental clinics, distributed the questionnaires to participants, and collected them upon completion. The questionnaires were self-administered. For each patient safety dimension, a percent positive score was first calculated at the item level using valid responses. Mean scores for each dimension were calculated by averaging item-level percent positive scores, resulting in values ranging from 0 to 1 (equivalent to 0% to 100%). For example, a mean score of 0.78 indicates that 78% of responses to items in that dimension were positive.

Finally, the overall MOSPSC composite score was calculated as the mean of all dimension scores. A positive patient safety culture was defined as a composite score above 60%. Dimensions scoring ≥75% were considered strengths, while those scoring <60% were considered weaknesses requiring improvement. For overall quality and patient safety assessment, the frequency and percentage of positive responses (based on valid responses) were computed. The same method was applied to each quality item: patient-centeredness, effectiveness, timeliness, efficiency, and equity. It is important to note that negatively worded items were reverse-scored [17].

Participants’ demographic characteristics and the central tendency and dispersion of the study variables were analyzed using descriptive statistics. Inferential analyses, including independent t-tests and analysis of variance (ANOVA), were conducted to examine associations between demographic variables and patient safety culture. Data were analyzed using SPSS (version 23), and the threshold for statistical significance was set at p < 0.05.

Multiple linear regression analysis (enter method) was performed to examine the association between demographic characteristics and the total patient safety culture score. Age, gender, work experience (<6 vs. ≥ 6 years), weekly work hours (≤40 vs. > 40), job position (dentist vs. other), and managerial position (yes/no) were entered simultaneously into the final model. Education was excluded because of severe multicollinearity with job position. Model assumptions were assessed before analysis. Residuals were approximately normally distributed, and no evidence of problematic multicollinearity was observed in the final model (all VIFs < 5).

### Ethical considerations

The study was approved by the Ethics Committee of Mashhad University of Medical Sciences, Iran (approval code: IR.MUMS.FHMPM.REC.1403.032), with Afghanistan specified as the study setting. Local engagement was facilitated by a co-author who was a dentist in Herat and a faculty member at Hariva University at the time of data collection. Hariva University was aware of and permitted the research activities. Permission to conduct the study was also obtained from the managers of the participating dental clinics. Informed consent was obtained from all study participants.

### Inclusivity in global research

Additional information regarding the ethical, cultural, and scientific considerations specific to inclusivity in global research is provided in the Supporting Information (S1 Checklist).

## Results

The average age of study participants was 33.66 ± 7.0 years. Most of the respondents were male (67.1%) and worked for 6–11 years at the institution (24.3%). The largest proportion of respondents, (47.6%) worked more than 40 hours per week. Moreover, 74.3% of the respondents were dentists. The majority of the respondents (63.8%) had a master’s degree and 17.6% held managerial positions.

There was no significant relationship between the total patient safety culture score and demographic characteristics, except for education, which showed a significant association (Table 1).

**Table 1:** Characteristics of study participants and relationship between the total patient safety culture score and demographic characteristics.

Variable	Mean ±SD	Min	Max	P-value
Age	33.66 ± 7.0	20	50	0.482^1^ ^r = 0.049^
	Categories	Number	Percentage	
Gender	Female	69	32.9	0.240^2^
	Male	141	67.1	
Years worked at primary location	6 > years	108	0.81	0.674^2^
	≥6 years	102	0.78	
Time worked at the clinic	Less than 2 months	6	2.9	0.686^3^
	2 months to a year	25	11.9	
	1-3 years	37	17.6	
	3-6 years	46	21.9	
	6-11 years	51	24.3	
	More than 11 years	45	21.4	
Work hours a week in the institution	1-4	14	6.7	0.956^3^
	5-16	38	18.1	
	17-24	14	6.7	
	25-32	26	12.4	
	33-40	18	8.6	
	40<	100	47.6	
Job	Dentist	156	74.3	0.956^2^
	Others	54	25.7	
Education	Dental student	4	1.9	0.034^3^
	Bachelor	51	24.3	
	Master	134	63.8	
	PhD in oral and maxillofacial surgery	13	6.2	
	Oral and maxillofacial surgery resident	8	3.8	
Managerial position	Yes	37	17.6	0.857^2^
	**No**	173	82.4	

^1^Pearson ^2^ T-test, ^3^ Variance Analysis.

As shown in Table 2, the surveyed dental clinics reported the highest mean percentage-positive scores in organizational learning (0.78 ± 0.14) and teamwork (0.77 ± 0.17) and the weakest in office processes and standardization (0.62 ± 0.13). Among the quality assessment items, effectiveness had the highest score (0.68 ± 0.46), and efficiency ranked lowest (0.48 ± 0.50). The score obtained for evaluating the overall clinical processes and systems for preventing, identifying, and correcting potential problems affecting patients was 0.66 ± 0.47, indicating a rating of ‘good’ or ‘very good.’

**Table 2:** Composite and Overall Quality and Safety Items in the participating dental clinics.

Culture dimensions	Mean ±SD	Number of participants*	Interpretation**
overall perceptions of patient safety and quality	0.68 ± 0.13	210	Strength
organizational learning	0.78 ± 0.14	210	Strength
Teamwork	0.77 ± 0.17	206	Strength
staff training	0.73 ± 0.16	206	Strength
work pressure and pace	0.72 ± 0.14	208	Moderate
management support for patient safety	0.71 ± 0.12	154	Moderate
office processes and standardization	0.62 ± 0.13	208	Moderate
communication about error	0.74 ± 0.17	210	Moderate
communication openness	0.70 ± 0.15	210	Moderate
patient care tracking/follow-up	0.73 ± 0.16	210	Moderate
Total patient safety culture score	0.70 ± 0.10	210	Moderate
Overall rating on quality items			
Patient centered	0.62 ± 0.11	210	Moderate
Effective	0.68 ± 0.46	210	Moderate
Timely	0.56 ± 0.49	210	Weakness
Efficient	0.48 ± 0.50	210	Weakness
Equitable	0.67 ± 0.47	210	Moderate
Overall Rating on patient safety			
Processes for preventing and correcting patient problems	0.66 ± 0.47	210	Moderate

*N ranged from 154 to 210 across dimensions due to missing responses. **Dimensions with positive scores ≥75% were considered strengths, while scores <60% were considered areas requiring improvement.

Regarding the frequency of patient safety events, the most favorable finding was related to patient identification, with 50.5% of participants reporting that this issue had not occurred in the past 12 months. In contrast, the weakest performance was observed for access to care, with 11.8% of participants reporting that patients were unable to obtain an appointment within 48 hours for an acute or serious problem on a daily basis. Additionally, 34.0% of respondents reported that problems in exchanging accurate, complete, and timely information with pharmacies had not occurred in the past 12 months. Overall, 65.7% of participants rated the clinical processes and systems for preventing, identifying, and correcting potential patient problems as good or very good (Table 3).

**Table 3:** Patient safety and quality issues in the studied clinics in the past year.

Time set/ event frequency (%)	Daily	Weekly	Monthly	Several times in the past 12 months	Once or twice in the past 12 months	Not in the past 12 months	Total
Access to Care	A patient was unable to get an appointment within 48 hours for an acute/serious problem						
	23(11.8%)	8(1.4%)	16(8.2%)	29(14.9%)	47(24.1%)	72(36.9%)	195(100%)
Patient Identification	The wrong chart/medical record was used for a patient.						
	3(1.5%)	10(5.0%)	7(3.5%)	32(16.0%)	47(23.5%)	101(50.5%)	200(100%)
Charts/Medical Records	A patient’s chart/medical record was not available when needed.						
	5(2.7%)	8(4.3%)	11(6.0%)	23(12.5%)	65(35.5%)	72(39.1%)	184(100%)
	Medical information was filed, scanned, or entered into the wrong patient’s chart/medical record						
	2(1.0%)	5(2.6%)	5(2.6%)	30(15.5%)	67(34.7%)	84(43.5%)	193(100%)
Medical Equipment	Medical equipment was not working properly or was in need of repair or replacement.						
	7(3.4%)	7(3.4%)	15(7.4%)	59(28.9%)	76(37.3%)	40(19.6%)	204(100%)
Medication	A pharmacy contacted our office to clarify or correct a prescription.						
	5(2.6%)	11(5.8%)	11(5.8%)	38(20.0%)	45(23.7%)	80(42.1%)	190(100%)
	A patient’s medication list was not updated during his or her visit.						
	6(3.2%)	10(5.3%)	23(12.3%)	46(24.6%)	48(25.7%)	54(28.9%)	187(100%)
**Diagnostics & Tests**	The results from a lab or imaging test were not available when needed.						
	2(1.0%)	7(3.6%)	10(5.2%)	31(16.1%)	68(35.2%)	75(38.9%)	193(100%)
	A critical abnormal result from a lab or imaging test was not followed up within 1 business day.						
	11(5.7%)	5(2.6%)	29(14.9%)	36(18.6%)	41(21.1%)	72(37.1%)	194(100%)
Problems exchanging accurate, complete, and timely information with							
Outside labs/imaging centers	17(8.9%)	9(4.7%)	31(16.3%)	52(27.4%)	33(17.4%)	48(25.3%)	190(100%)
Other dental offices/outside dentists	13(6.9%)	12(6.3%)	20(10.6%)	33(17.5%)	49(25.9%)	62(32.8%)	189(100%)
Pharmacies	15(7.6%)	24(12.2%)	20(10.2%)	16(8.1%)	55(27.9%)	67(34.0%)	197(100%)
Medical offices/outside physicians	15(7.5%)	16(8.0%)	31(15.6%)	37(18.6%)	45(22.6%)	55(27.6%)	199(100%)

As shown in Table 4, the overall model was statistically significant (F(6,203)=2.919, p = 0.009), although it explained only a small proportion of the variance in the total patient safety culture score (R² = 0.079). Among the demographic characteristics examined, only gender and managerial position were associated with the total patient safety culture score. Female participants and those holding managerial positions had significantly higher patient safety culture scores than male participants and non-managerial staff, respectively. No significant associations were observed for age, work experience, weekly work hours, or job position.

**Table 4:** Multiple linear regression results for total patient safety culture score (N = 210).

Variable	B	Std. Error	Standardized β	p-value	95% CI for B
(Constant)	0.652	0.061	–	<0.001	0.532–0.771
Age	0.001	0.002	0.047	0.634	−0.002–0.004
Gender (Female = 1)	0.031	0.016	0.134	0.049	0.000–0.062
Managerial position (Yes = 1)	0.073	0.020	0.258	<0.001	0.034–0.113
Work experience (<6 years = 1)	0.009	0.019	0.042	0.636	−0.029–0.047
Weekly work hours (≤40 = 1)	−0.004	0.019	−0.017	0.841	−0.040–0.033
Job position (other = 1)	0.018	0.022	0.073	0.418	−0.026–0.062

Note: Model F (6, 203) = 2.919, p = 0.009, R^2^ = 0.079, Adjusted R^2^ = 0.052. Dependent variable: total patient safety culture score (range 0–1)

## Discussion

According to the results of the present study, dental institutions in Herat, Afghanistan, exceeded the predefined acceptable threshold (60%) for overall patient safety culture in 2024. The highest mean percentage-positive scores were for organizational learning and teamwork, while the lowest mean percentage-positive score was observed for office processes and standardization. In Afghanistan, few studies have examined patient safety culture in the healthcare settings, and none, to our knowledge have addressed dentistry. In one governmental hospital study, the overall safety culture score was low [3]; however, in the dental institutions examined in the present study, it was at a moderate level.

This difference should be interpreted cautiously because the two studies involved different healthcare settings, professional groups, and organizational contexts; therefore, the higher score observed in dental institutions should not be interpreted as evidence that dental care is inherently safer than hospital care. Rather, it may reflect differences in the characteristics and organization of outpatient dental services.

In addition, among quality items in this study, patient-centeredness, effectiveness, and equity were at moderate levels, whereas timeliness and efficiency were below the predefined threshold. More than half of the participants stated that most listed safety-related incidents either did not occur at all or occurred only once or twice in the past year. Additionally, a substantial number of study participants rated their centers’ processes and clinical systems for preventing, detecting, and correcting potential problems as very good or excellent. The overall rating of processes for preventing, identifying, and correcting potential patient problems was relatively favorable, aligning with findings from studies conducted in Iran [18] and Spain [8]. In Finland, about 35% of dentists reported some patient safety issues in the previous year. Nearly one-third of adverse incidents involved dental equipment and supplies, and about one-tenth were likely to cause or did cause permanent harm, highlighting the importance of standardization and equipment maintenance [19].

A study on patient safety culture in US dental schools showed the highest performance in teamwork and the lowest in “patient care and catch patient problems” and management support for patient safety.” Moreover, all aspects of patient safety in dentistry received lower scores than medical practice [20].In another U.S. study comparing patient safety culture between dentistry and medicine, dental staff positively rated teamwork and patient follow-up but reported low safety scores for handoffs and communication errors, revealing significant gaps in patient safety culture [11]. Another U.S. study, “Lessons learned from dental patient safety case reports,” found that delayed or unnecessary treatment and disease progression after misdiagnosis were the most common types of harm. Of the cases reviewed, 24.4% of patients experienced permanent harm from an adverse event, and one in ten (11.1%) ended in death [21].

Taken together, these findings suggest that favorable perceptions of some organizational dimensions do not necessarily imply the absence of clinically important safety risks. In dental care, relatively infrequent but potentially serious incidents may remain difficult to detect through routine safety-culture surveys, particularly when reporting systems are limited. This distinction is important when interpreting the moderate overall score in the present study.

The studied institutions had difficulty exchanging accurate, complete, and timely information, especially with outside labs (e.g., medical and pathology labs). The mean positive score for “communication about errors” was at a moderate level. In Tehran, Iran teaching hospitals, the lowest score was in “exchanging and sharing data.” In addition, logistic regression results showed that communication, error feedback, and information transfer significantly predicted adverse events [22]. A systematic review on patient safety culture reported low communication scores [23]. Moreover, the observed scores for communication openness were consistently above the predefined threshold, indicating a moderate level of patient safety performance, whereas a Spanish tertiary hospital study reported the lowest scores in this dimension [8].

The moderate communication openness observed within clinics contrasts with difficulties in exchanging information with external laboratories, suggesting that internal communication may be more developed than inter-organizational communication. Since dental care often depends on accurate information from external providers, these findings highlight the importance of reliable information transfer across organizational boundaries for patient safety.

The score for office processes and standardization was above the predefined 60% threshold but remained the weakest dimension, indicating a moderate level of performance. This finding is consistent with studies from Iranian hospitals, which also reported moderate scores for this dimension [24,25]. A study on adverse incidents in dental care found that most events went unreported and unpublished, highlighting the importance of documentation and open communication about errors [9]. Understanding error types and causes is essential for improving patient safety and should be prioritized alongside process standardization.

This dimension’s weak performance highlights a practical organizational gap beyond what the moderate overall score implies. Standardizing documentation, patient records, incident reporting, and follow-up may improve consistency and error detection.

Organizational learning scored high, consistent with studies from the US, Iran, Afghanistan, and a systematic review [3,18,22–24]. However, a high score should not be equated with well-established formal learning systems, especially where incident reporting is limited. In Finland, greater dentist awareness of patient safety risks improved their ability to identify harmful situations. Patient complaints and case reports can be used for learning [26]. In the present study, the training dimension also received favorable scores. These strengths provide a useful foundation for addressing weaker areas such as standardization, follow-up, and information exchange.

Management support for patient safety was above the minimum threshold, similar to two Iranian studies [18,24], in contrast with the review by Reis et al. [23] and the Spanish study [8]. Without active and visible leadership engagement, efforts to improve patient safety culture are unlikely to succeed. Visible managerial support and a non-punitive environment may facilitate error reporting and support improvements in patient safety culture. The moderate rather than clearly strong level of management support in the present study also suggests that organizational leadership may be an important area for further improvement, particularly in supporting standardized processes and non-punitive reporting.

Patient care tracking/follow-up was relatively favorable, whereas studies in Iran [24,25] and Reis et al. reported lower scores [23]. This discrepancy may relate to the type of centers studied: previous research focused on hospitals and medical practice, while the present study examined dental clinics. Patient follow-up is crucial for early complication detection, timely treatment adjustments, and better health outcomes. Nevertheless, because patient follow-up was not the lowest-scoring dimension in the present study, it should be considered a secondary improvement priority rather than the primary weakness. Its importance lies particularly in its relationship with the weaker areas of office processes and information management.

Teamwork was rated favorably by the study participants. A U.S. study reported the highest positive scores for teamwork in dental practices [11]. Many studies also found highest scores for teamwork [8,24,25]. Effective teamwork may help reduce medical errors, improves patient outcomes, and enhances care quality [27]. Teamwork is an important component of a strong patient safety culture. When healthcare professionals collaborate effectively, they are better able to identify potential errors, communicate concerns openly, and ensure consistent adherence to safety protocols.

Participants reported a relatively favorable perception of work pressure and pace. In contrast to some hospital-based studies where staffing constraints and workload have been associated with adverse events [18,28], workload was not a prominent perceived weakness in the present study. This may reflect differences between dental and hospital settings. Teamwork and organizational learning scored highly in the present study and were also reported as relatively strong dimensions in previous studies [11,24,25]. In contrast, standardization, communication across organizational boundaries, and management support were comparatively weaker areas in the present study.

In the bivariate analyses, education was the only demographic characteristic associated with the total patient safety culture score. Similar associations between demographic characteristics and patient safety culture have been reported in previous studies, although the specific associated variables have varied across settings [29,30]. This association was not retained in the multivariable analysis.

The present study showed that demographic characteristics explained only a small proportion of the variation in patient safety culture. Although gender and managerial position were independently associated with the total patient safety culture score, the overall explanatory power of the model was low. Similar findings have been reported in primary healthcare settings in Kuwait and public hospitals in Ethiopia, where demographic characteristics explained only a small proportion of the variability in patient safety culture [29,31].

These findings indicate that factors beyond the demographic characteristics examined in this study are likely to contribute to variation in patient safety culture. These results also reinforce the importance of avoiding an overly individual-level interpretation of patient safety culture. Because most of the variation remained unexplained by staff characteristics, organizational factors—such as leadership practices, reporting systems, workflow design, communication processes, and availability of standardized procedures—may warrant greater attention in future research.

Female participants reported higher patient safety culture scores than male participants. Previous studies have reported inconsistent findings, with some demonstrating higher safety culture scores among women, whereas others found higher scores among men or no independent association after adjustment for other factors [30,32,33].

Participants in managerial positions also reported higher patient safety culture scores, consistent with findings from previous studies showing that managers generally perceive patient safety culture more positively than frontline staff [33]. This may reflect managers’ closer involvement in quality improvement activities, greater awareness of organizational safety initiatives, or a more optimistic assessment of safety practices due to their oversight role. Accordingly, the difference between managerial and non-managerial perceptions should be considered when planning improvement initiatives, and frontline staff perspectives should be incorporated into safety assessments and quality-improvement activities.

### Policy and practical implications

The findings have implications for both dental clinics and health policymakers in Herat. At the clinic level, improvement efforts should prioritize office processes and standardization, particularly patient information recording, documentation, incident reporting, and patient follow-up. Clinics should also strengthen the timely and accurate exchange of information with outside laboratories and other healthcare providers. Regular patient safety training and continued managerial support may help maintain the strengths observed in teamwork and organizational learning while addressing these weaker areas. At the policy level, relevant health authorities and professional organizations could develop and promote standardized patient safety procedures, documentation and incident-reporting systems, and follow-up processes that are feasible within the available resources and organizational context of dental services in Afghanistan.

### Limitations and strengths

This study is among the few studies addressing patient safety culture in dental settings and, to our knowledge, is the first in Afghanistan. A well-established instrument was used, enhancing the reliability and comparability of the findings. However, patient safety culture was assessed based on staff perceptions. Therefore, the findings may not fully reflect actual safety practices or patient safety incidents in the dental clinics. As the study was conducted in dental institutions in Herat, the findings should be generalized to other dental settings in Afghanistan with caution. Additionally, the multiple regression model explained only 7.9% of the variance in patient safety culture scores (R² = 0.079), indicating that other unmeasured factors may contribute substantially to variation in patient safety culture.

## Conclusion

This study provides the first empirical evidence that patient safety culture in dental institutions of Herat, Afghanistan, was at a moderate level and exceeded the predefined 60% threshold. The strongest domains were organizational learning and teamwork, while the lowest-scoring patient safety culture dimension was office processes and standardization.

Policymakers and clinic managers should prioritize office processes and standardization, timeliness, and efficiency, while strengthening patient follow-up and information exchange with external providers. These efforts should build on the existing strengths in teamwork and organizational learning and consider the local organizational and resource context.

## Supporting information

S1 Checklist Inclusivity in global research questionnaire. Completed questionnaire addressing ethical, cultural, and scientific considerations specific to inclusivity in global research.
(DOCX)
