# Supplementary material for: Patient safety culture in dental clinics: A cross-sectional study in Afghanistan
Source: PLoS One. 2026 Oct 28;21(10):e0359289. doi: 10.1371/journal.pone.0359289 (PMC13716210; doi:10.1371/journal.pone.0359289)
Supplement: Supplementary file 1 [file pone.0359289.s001.docx]

Inclusivity in global research

PLOS’ policy on inclusivity in global research aims to improve transparency in the reporting of research performed outside of researchers’ own country or community and ensures that PLOS publications reporting global research adhere to high standards for research ethics and authorship. Authors of relevant research articles may be asked to complete the questionnaire below, which outlines ethical, cultural, and scientific considerations specific to inclusivity in global research. This questionnaire may be requested when researchers have travelled to a different country to conduct research, if research uses samples collected in another country, research with Indigenous populations or their lands, or if research is on cultural artefacts. Researchers travelling to another country solely to use laboratory equipment will not normally be required to complete the questionnaire. However, the questionnaire can be requested at the journal’s discretion for any submission – if you have been requested to complete this questionnaire by the PLOS journal you submitted to, please do so.

Please complete the questionnaire below and include this as a Supporting Information file with your manuscript. Note that if your paper is accepted for publication, this checklist will be published with your article in the supporting information files. Please ensure that you reference the checklist in the main body of your manuscript. We suggest adding a subsection ‘Inclusivity in global research’ to your Methods section and adding the following sentence: “Additional information regarding the ethical, cultural, and scientific considerations specific to inclusivity in global research is included in the Supporting Information (SX Checklist)”

The questions have been designed to be applicable to a wide range of study types, and there are subsections for both human subjects research and non-human subjects research. If any of the questions are not relevant to your research please mark them as “N/A” as appropriate.

**Ethical considerations, permits and authorship**

*This section is applicable to all research types.*

Provide details as to who granted permissions and/or consent for the study to take place in the Methods section of your manuscript. This should include the names of **all** ethics boards, governmental organizations, community leaders or other bodies that provided approval for the study. If individuals provided approval refer to these people by their role or title but do not list their name(s).

Reported on page number: 10

The study was approved by the Ethics Committee of Mashhad University of Medical Sciences, Iran, with Afghanistan specified as the study setting. Local engagement was facilitated by a co-author who was a dentist in Herat and a faculty member at Hariva University at the time of data collection. Hariva University was aware of and permitted the research activities. Permission to conduct the study was also obtained from the managers of the participating dental clinics. Informed consent was obtained from all study participants.

If there were any deviations from the study protocol after approval was obtained please provide details of these changes in the Methods section of your manuscript.

Reported on page number: NA

No deviations from the approved study protocol were reported.

Did this study involve local collaborators that are residents of the country where the research was conducted or members of the community studied? If you do not have any authors from said communities, please provide an explanation for this below.

Yes. A co-author was a dentist in Herat and a faculty member at Hariva University during data collection. This local collaborator facilitated local engagement and coordination during the study.

Everyone listed as an author should meet PLOS’ criteria for authorship and all individuals who meet these criteria should be included in the author byline, rather than the acknowledgements. For further information please see the journal’s Authorship Policy.

**Human subjects research (e.g. health research, medical research, cross-cultural psychology)**

Did you obtain written informed consent from a representative of the local community or region before the research took place? How did you establish who speaks for the community? Details of written informed consent obtained from study participants should be reported separately in the Methods section of your manuscript.

No. No separate written informed consent was obtained from a formally designated representative of the local community or region. Local engagement was facilitated by a co-author who was a dentist in Herat and a faculty member at Hariva University during data collection. Permission to conduct the study was obtained from the managers of the participating dental clinics. Informed consent was obtained from individual study participants.

How did members of the local community provide input on the aims of the research investigation, its methodology, and its anticipated outcome(s)?

Input was obtained through a pilot study involving 30 dental staff members from Herat, which assessed the appropriateness and clarity of the translated questionnaire. A co-author who was a dentist in Herat and a faculty member at Hariva University during data collection also provided local input during the research process.

When engaging with the local community, how did you ensure that the informed consent documents and other materials could be understood by local stakeholders?

The questionnaire and informed consent statement were provided in Dari, the commonly understood language among dental staff in Herat. The consent statement used simple and clear language. A pilot study with 30 participants confirmed that the materials were understandable, and participants could ask questions before completing the questionnaire.

Will the findings of the research be made available in an understandable format to stakeholders in the community where the study was conducted (e.g. via a presentation, summary report, copies of publications, etc.)? Please provide details of how this will be achieved.

Yes. A summary of the findings will be prepared in Dari and shared with participating dental clinics and Hariva University. The published article will also be made available to stakeholders upon request.

**Non-human subjects research using specimens/ animals collected as part of the study, or those housed in archival collections. Examples include archaeology, paleontology, botany and zoology.**

Did the permission you obtained from a local authority to perform the study include an agreement on access to outputs and benefit sharing? This may include procedures to enable fair distribution of the benefits and resources arising from the research performed. Please include any details of Prior Informed Consent and Benefit Sharing Agreements obtained. These may be required by field-specific regulations, for example the Convention on Biological Diversity (CBD) and the associated Nagoya Protocol.

N/A — This study did not involve specimens, animals, archaeological materials, or cultural artifacts. It was a survey-based study on human subjects (dental staff).

If the material used in your study was imported, please A) provide the year it was imported and B) indicate whether permits were obtained to import/export the materials used, C) provide details of any permits obtained. If this information is not available, please indicate this.

N/A — No materials were imported for this study.

If you used archival specimens, please state how the material used in your study was acquired by the institute it is held in and provide details of any permits obtained for the original excavations/ sample collection. If this information is not available, please indicate this.

N/A — No archival specimens were used in this study.

How was the potential cultural significance of the materials collected in your study to local communities considered in your research design? Were Indigenous peoples and/or local researchers and institutions involved with archaeological excavations / collection of specimens? If so, please provide a description of their involvement.

N/A — This study did not involve collection of any physical materials, specimens, or cultural artifact

If your manuscript includes photographs of human remains please indicate whether authors obtained permission from descendants or affiliated cultural communities to do so.

N/A — No photographs of human remains are included in this manuscript.
