# Supplementary material for: Patient safety culture in dental clinics: A cross-sectional study in Afghanistan
Source: PLoS One. 2026 Oct 28;21(10):e0359289. doi: 10.1371/journal.pone.0359289 (PMC13716210; doi:10.1371/journal.pone.0359289)
Supplement: Supplementary file 2 [file pone.0359289.s003.docx]

Dear Dr. Yolanda Malele-Kolisa (Academic Editor),

Thank you for your valuable comments and for the opportunity to revise our manuscript entitled "Patient Safety Culture in Dental Clinics: A Cross-sectional Study in Afghanistan" (PONE-D-26-31020). We have carefully addressed all the points raised by the reviewers and the editor. Below, we provide our point-by-point responses.

| Comment | Response |
| --- | --- |
| Reviewer 1 |  |
| Correct typos in the document | Thank you for this comment. We have carefully reviewed the entire manuscript and corrected all typographical errors. Specific corrections have been made to lines 97 and 98 as indicated. The revised manuscript has been thoroughly proofread. |
| Line 98: suggest replacing dentists with dental practitioners to include both dental hygienists and students. | Thank you for this comment. We have replaced "dentists" with "dental practitioners" in line 98 as suggested. |
| Be sure to: Add policy and practical implications in your recommendations. | Thank you for this comment. We have added specific policy and practical implications in the Discussion and Conclusion sections. These include concrete recommendations for policymakers, clinic managers, and future research. |
| Reviewer 2 |  |
| The reporting of regression analyses should be more comprehensive, including the presentation and interpretation of key statistical parameters. | Thank you for this comment. We have revised the reporting of the multiple linear regression analysis to provide a more comprehensive presentation and interpretation of the key statistical parameters. The revised manuscript now reports the regression coefficients (B), standard errors, standardized coefficients (β), p-values, and 95% confidence intervals for all variables, as well as the overall model F-statistic, R², and adjusted R². We have also clarified the interpretation of the model, noting that it was statistically significant (F(6,203)=2.919, p=0.009) but explained a relatively small proportion of the variance in patient safety culture scores (R²=0.079; adjusted R²=0.052). The Discussion further explains the implications of this limited explanatory power and the potential contribution of unmeasured organizational factors. |
| The introduction would benefit from a stronger contextual background that better highlights the importance of patient safety culture in dental healthcare settings. | Thank you for this comment. We have strengthened the introduction by adding more context on the importance of patient safety culture specifically in dentistry, and the gap in knowledge in Afghanistan. The revised introduction now better establishes the rationale for this study. |
| The discussion section should provide deeper engagement with relevant literature and offer a more critical interpretation of the findings. | Thank you for this comment. We have substantially revised the Discussion section by adding deeper comparisons with findings from relevant studies conducted in different countries (including the US, Iran, spain, and Finland), providing more critical and context-sensitive interpretations of the observed differences across patient safety culture dimensions, and clarifying the policy and practical implications of the findings. |
| Clarity is also needed regarding the interpretation and practical implications of the reported patient safety culture scores. Readers would benefit from a clearer explanation of what the scores indicate and how they compare with findings from other settings. | We have clarified the interpretation of scores. We now clearly explain that a score of 0.70 indicates a moderate level, with strengths in organizational learning (0.78) and teamwork (0.77), while office processes and standardization had the lowest score (0.62) among the patient safety culture dimensions. Comparisons with other settings have been added. |
| The manuscript should explicitly acknowledge its limitations, including any methodological constraints that may affect the generalizability of the results. | Thank you for this comment. We have explicitly acknowledged the key methodological limitations of the study in the revised Limitations and Strengths section. These include the reliance on staff perceptions, which may not fully reflect actual safety practices or patient safety incidents, the single-city setting, which warrants caution when generalizing the findings to other dental settings in Afghanistan, and the low explanatory power of the regression model (R²=0.079), indicating that other unmeasured factors may contribute substantially to variation in patient safety culture. |
| requirements |  |
| PLOS ONE Style Requirements | We have carefully reviewed and revised our manuscript to ensure compliance with PLOS ONE's style requirements. |
| Inclusivity in Global Research Questionnaire | We have completed the "Inclusivity in Global Research" questionnaire and have uploaded it as Supporting Information (S1 Checklist). We have also added a subsection titled "Inclusivity in global research" in the Methods section of the manuscript, with the following sentence: |
| Data Availability Statement | We modified it. All relevant data are within the manuscript and its supporting Information files. |
| **ORCID ID for Corresponding Author** | We confirm that the corresponding author (Dr. Fatemeh Kokabisaghi) has a verified ORCID iD (0000-0002-9745-8784) in the Editorial Manager system. The ORCID iD has been validated following the instructions provided. |
| **Ethics Statement Location** | We have moved the ethics statement from the end of the manuscript to the **Methods section**. It is now placed under a dedicated subsection titled "Ethical considerations.  The statement has been removed from its previous location.  The following text has been added to the Methods section: |
| **Supporting Information Captions** | We have added captions for all Supporting Information files at the end of the manuscript and have updated in-text citations accordingly. The following caption has been included:  **Supporting Information:**  **S1 Checklist. Inclusivity in global research questionnaire.** Completed questionnaire outlining ethical, cultural, and scientific considerations specific to inclusivity in global research, as required by PLOS ONE policy. |
| **Reference List Review** | We have thoroughly reviewed our reference list to ensure all citations are complete, correct, and current. No retracted articles were identified in our reference list. All references have been verified and formatted according to PLOS ONE style requirements. |

We have also:

1. Moved the ethics statement from the end of the manuscript to the Methods section

2. Added an "Inclusivity in global research" subsection in Methods with a Supporting Information checklist

3. Corrected inconsistencies between Abstract, Results, and Conclusion regarding significant variables

4. Standardized terminology throughout the manuscript

We believe these revisions have substantially improved the manuscript. We are grateful for the reviewers' constructive feedback.

Sincerely,

Corresponding Author:

Dr. Fatemeh Kokabisaghi

Email: Kokabisaghi.f@gmail.com
